# Prevalence of Colistin-Resistant Bacteria among Retail Meats in Japan

**DOI:** 10.14252/foodsafetyfscj.D-21-00002

**Published:** 2021-06-25

**Authors:** Justice O. Odoi, Sayo Takayanagi, Michiyo Sugiyama, Masaru Usui, Yutaka Tamura, Tetsuo Asai

**Affiliations:** 1 Department of Applied Veterinary Sciences, United Graduate School of Veterinary Sciences, Gifu University, 1-1 Yanagido, Gifu 501-1193, Japan.; 2 Faculty of Applied Biological Sciences, Gifu University, 1-1 Yanagido, Gifu 501-1193, Japan.; 3 Laboratory of Food Microbiology and Food Safety, School of Veterinary Medicine, Rakuno Gakuen University, 582 Midorimachi, Bunkyodai, Ebetsu 069-8501, Japan.; 4 Education and Research Center for Food Animal Health, Gifu University (GeFAH), 1-1 Yanagido, Gifu 501-1193, Japan.

**Keywords:** colistin, gram-negative bacteria, plasmid-mediated colistin-resistance gene, retail meat

## Abstract

Colistin (CST) is considered the last resort for the treatment of infectious diseases due to multidrug-resistant bacteria. Since the *mc*r-1 gene has been reported in *Enterobacteriaceae* isolated from food, animals, and humans in China, the prevalence of CST-resistant bacteria has been of great concern. Here, we investigated the prevalence of CST resistance and plasmid-mediated colistin-resistance genes (*mcr*) in gram-negative bacteria isolated among retail meats in Japan. CST-resistant bacteria were isolated from 310 domestic retail meats (103 chicken meat, 103 pork, and 104 beef) purchased between May 2017 and July 2018 from retail shops in Japan using CST-containing media and antimicrobial susceptibility testing. The *mcr* gene was investigated in isolates with a CST minimum inhibitory concentration of ≥1 μg/mL. Excluding the intrinsically CST-resistant isolates, CST-resistant bacteria were isolated from 39 of the total chicken meats (37.9%), 19 of the pork samples (18.4%), and 18 of the beef samples (17.3%). A total of 459 isolates were identified, out of which 99 were CST-resistant. CST resistance (resistance breakpoints: *Aeromonas*, >4 μg/mL; others, >2 μg/mL) was found in *Aeromonas* spp. (48/206, 23.3%), *Yersinia* spp. (5/112, 4.5%), *Escherichia coli* (23/39, 59%), *Citrobacter* spp. (4/26, 15.4%), *Klebsiella* spp. (2/23, 8.7%), *Raoultella* spp. (2/16, 12.5%), *Enterobacter* spp. (7/14, 50%), *Pseudomonas* spp. (1/8, 12.5%), *Pantoea* spp. (5/7, 71.4%), *Ewingella* spp. (1/4, 25%), and *Kluyvera* spp. (1/2, 50%). The *mcr* gene was detected in 16 isolates: *mcr*-1 in 14 isolates of *E. coli* from 10 chicken samples (9.7%), and *mcr*-3 in two isolates of *Aeromonas sobria* from pork and chicken samples (each 1.0%). The findings of this study highlight the necessity of surveillance of CST resistance and resistance genes in bacteria that contaminate retail meats.

## 1. Introduction

Colistin (CST), a critically important antimicrobial agent, is considered the last resort for the treatment of infections due to multidrug-resistant bacteria^1^^)^. CST acts on gram-negative bacteria by targeting lipopolysaccharides (LPS) on the outer membrane, leading to cell wall disintegration and subsequent bacterial death^2^^)^. The resistance mechanism against CST is likely due to chromosomal mutations in the two-component system, such as PhoPQ and PmrAB, located on the chromosome of gram-negative bacteria, leading to the modification of LPS through the reduction of the negative charge of the lipid A moiety^3^^)^. An alternate mechanism involves the plasmid-mediated CST-resistance gene (*mcr*). The *mc*r-1 gene found in *Enterobacteriaceae* isolated from food animals and humans was recently reported in China^4^^)^. Thereafter, a surveillance study conducted in Denmark identified the *mcr*-1 gene in 0.2% of *Escherichia coli *(*E. coli*)** implicated in human bloodstream infections^5^^)^. In addition to *mcr*-1, other *mcr* genes including *mcr-*2^6^^)^, *mcr*-3^7^^)^, *mcr*-4^8^^)^, *mcr*-5^9^^)^, *mcr*-6^10^^)^, *mcr*-7^11^^)^, *mcr*-8^12^^)^, *mcr*-9^13^^)^, and *mcr*-10^14^^)^ have been found in different plasmids in gram-negative bacteria.

The *mcr* genes have been frequently found in *Enterobacteriaceae* isolated from chickens^15^^)^, pigs^16^^)^, and turkeys^17^^)^. Additionally, the *mcr* gene has been identified in other bacteria, including *Aeromonas* spp.^18^^)^, *Acinetobacter* spp., and *Pseudomonas* spp.^19^^)^. In Japan, the *mcr*-1 gene has been detected in *E. coli* isolated from livestock and humans^20^^,^^21^^,^^22^^)^. The prevalence of *mcr*-1 (30%), *mcr*-3 (8.3%), and *mcr*-5 (28.3%) in *E. coli* was reported among diseased pigs^23^^)^. Moreover, the prevalence of the *mcr-1* gene in *E. coli* isolated among retail meats was also evaluated^24^^)^. These reports prompted the Food Safety Commission of Japan (FSCJ) to undertake a risk assessment of CST use in food-producing animals in 2017^25^^)^. Results of this assessment revealed a medium risk, and as a result, the Ministry of Agriculture, Forestry and Fisheries of Japan issued a directive for the withdrawal of CST as a growth promoter and relegated the drug as a second-choice antimicrobial agent for therapeutic use in food-producing animals. In the assessment, the FSCJ also recommended that important information on the prevalence of *mcr* genes in bacteria be regularly updated to guide necessary interventions for infection control. Therefore, in this study, we investigated the prevalence of CST resistance and *mcr* genes in gram-negative bacteria isolated among retail meats in Japan.

## 2. Materials and Methods

### Sample Collection of Retail Meats

A total of 310 domestic retail meats comprising 103 chicken meats, 103 pork samples, and 104 beef samples were purchased between May 2017 and July 2018 from retail shops in Hokkaido (33 samples), Iwate (27 samples), Tokyo (46 samples), Chiba (24 samples), Gifu (60 samples), Shiga (24 samples), Osaka (36 samples), Fukuoka (30 samples), and Kumamoto (30 samples) prefectures located in Japan.

### Isolation and Identification of Bacteria among Retail Meats

Using sterile forceps, each meat sample was gently pressed against the surface of deoxycholate hydrogen sulfide lactose (DHL) agar supplemented with 0.1 μg/mL of CST (CST-DHL medium) and incubated at 37°C overnight. For enrichment isolation, 5 g of each meat sample was aseptically cut and first cultured in 45 mL of tryptic soy broth (enrichment medium) at 37°C overnight. Afterward, the obtained bacterial suspension was streaked onto the CST-DHL medium and incubated at 37°C overnight. A maximum of three distinct red colonies formed per sample were preferentially picked at random. The properties of the bacteria were examined using triple sugar iron medium, lysine indole motility medium (Eiken Chemical Co., Ltd., Tokyo, Japan), and cytochrome oxidase test filter paper (Nissui Pharmaceutical Co., Ltd., Tokyo, Japan). The isolates were analyzed using VITEK^®^ 2 GN identification card (Sysmex BioMérieux, Tokyo, Japan) and PCR to further identify *Aeromonas* spp., as described previously^26^^)^.

### Antimicrobial Susceptibility Testing

The minimum inhibitory concentration (MIC) of antimicrobials for bacteria isolated among retail meats were determined by the broth microdilution method using a frozen plate (Eiken Chemical Co., Ltd.,) following the manufacturer’s instructions. The following 15 antimicrobial agents were tested: CST (0.25–8 μg/mL), ampicillin (AMP, 2–64 μg/mL), cefazolin (CFZ, 1–32 μg/mL), cefotaxime (CTX, 0.5–16 μg/mL), ceftazidime (CAZ, 1–32 μg/mL), meropenem (MEM, 0.5–16 μg/mL), tetracycline (TET, 2–64 μg/mL), gentamicin (GEN, 2–64 μg/mL), kanamycin (KAN, 4–128 μg/mL), amikacin (4–128 μg/mL), nalidixic acid (NAL, 4–128 μg/mL), ciprofloxacin (CIP, 0.12–4 μg/mL), levofloxacin (0.25–8 μg/mL), chloramphenicol (CHL, 4–128 μg/mL), and sulfamethoxazole/trimethoprim (SXT, 9.5/0.5–152/8 μg/mL). The antimicrobial resistance breakpoints, except that for CST, were interpreted following the Clinical and Laboratory Standard Institute guidelines^27^^)^. The European Committee on Antimicrobial Susceptibility Testing resistance breakpoints were used for CST^28^^)^. Isolates resistant to three or more antimicrobial classes were identified as multi-drug resistant^29^^)^.

### Detection of the *mcr* gene

The presence of *mcr*-1 through *mcr*-5 genes was investigated in 365 isolates (except in intrinsically CST-resistant bacterial isolates) among retail meats with CST MICs of ≥1 μg/mL using primers, as described previously^6^^,^^7^^,^^8^^,^^9^^,^^30^^)^.

### Whole-genome Sequencing

The isolates harboring the *mcr* gene were analyzed by whole-genome sequencing. Whole-cell DNA was purified from each isolate using a QIAquick PCR purification Kit (QIAGEN, Hilden, Germany). A DNA sequencing library (insert size: 750–1,000 bp) was prepared using a QIAseq FX DNA Library Kit (QIAGEN) for paired-end sequencing on an Illumina MiSeq or iSeq sequencer (Illumina, Inc., San Diego, CA, USA) according to the manufacturer’s instructions. The whole-genome sequence data of each isolate was analyzed using whole-genome and plasmid sequence databases from GenEpid-J (https://gph.niid.go.jp/genepid-j/) and PlasmidFinder version 2.0.1 (https://cge.cbs.dtu.dk/services/PlasmidFinder/) to identify plasmid replicon type, and ResFinder version 4.1 to identify acquired antimicrobial resistance genes, which is available in the homepage of the Center for Genomic Epidemiology website (http://www.genomicepidemiology.org/). Briefly, to identify resistance genes on plasmid, the plasmid sequence data was extracted from the whole genome sequence with PlasmidFinder and analyzed using the nucleotide Basic Local Alignment Sequencing Tool (BLASTn; National Center for Biotechnology Information) against reference sequences for confirmation. By using the ResFinder version 4.1, we then searched for resistance genes in the plasmid sequence data as previously described^31^^)^.

## 3. Results

### Isolation of Bacteria among Retail Meats

A total of 1,125 bacteria were isolated from 281 of 310 retail meat samples (90.6%) purchased. Four hundred and fifty-four isolates were obtained from chicken samples (98/103, 95.1%), 348 isolates from pork samples (95/103, 92.2%), and 323 isolates from beef samples (88/104, 84.6%). In total, 18 bacterial genera, including six genera (*Cedecea* spp., *Hafnia* spp., *Morganella* spp., *Proteus* spp., *Providencia* spp., and *Serratia* spp.) that are intrinsically CST-resistant were isolated (data on intrinsically CST-resistant isolates not shown). Excluding the intrinsically CST-resistant isolates, the predominant genus isolated was *Aeromonas* spp. (206/459, 44.9%), followed by *Yersinia* spp. (112/459, 24.4%), and *Escherichia* spp. (39/459, 8.5%). The remaining bacteria belonged to *Citrobacter* spp., *Klebsiella* spp., *Raoultella* spp., *Enterobacter* spp., *Pseudomonas* spp., *Pantoea* spp., *Ewingella* spp., *Acinetobacter* spp., and *Kluyvera* spp. (Table 1).

**Table 1.** Colistin-resistant bacteria isolated among retail chicken meat, pork, and beef

Bacteria		Total pos. sample (Total isolates)	Chicken			Pork			Beef		
Genus	Species		Pos. sample (Isolate No.)	Pos. CST-res sample (CST-res Isolate No.)	% CST- res Isolates	Pos. sample (Isolate No.)	Pos. CST-res sample (CST-res Isolate No.)	% CST-res Isolates	Pos. sample (Isolate No.)	Pos. CST-res sample (CST-res Isolate No.)	% CST- res Isolates
*Aeromonas*	*sobria*	63(117)	50(101)	5(7)	6.9	10(13)	2(2)	15.4	3(3)	1(1)	33.3
	*hydrophila*	59(74)	38(44)	20(23)	52.2	14(18)	6(6)	33.3	7(12)	4(6)	50
	*veronii*	12(15)	12(12)	2(2)	16.7	2(2)	1(1)	50	1(1)	0	0
	Subtotal	98(206)	65(157)	27(32)	20	23(33)	8(9)	27.3	10(16)	5(7)	43.8
*Yersinia*	*enterocolitica*	57(100)	6(10)	0	0	22(34)	1(2)	5.9	29(56)	2(3)	5.4
	*frederiksenii*	7(9)	2(2)	0	0	4(6)	0	0	1(1)	0	0
	*intermedia*	3(3)	0	0	0	0	0	0	3(3)	0	0
	Subtotal	63(112)	8(12)	0	0	24(40)	1(2)	5	31(60)	2(3)	5
*Escherichia*	*coli*	27(39)	19(27)	13(19)	70.4	4(5)	2(2)	40	4(7)	1(2)	28.6
*Citrobacter*	*freundii*	14(15)	7(7)	0	0	3(4)	0	0	4(4)	2(2)	50
	*braakii*	6(9)	1(1)	0	0	3(5)	1(1)	20	2(3)	1(1)	33.3
	*amalonaticus*	1(2)	0	0	0	0	0	0	1(2)	0	0
	Subtotal	21(26)	8(8)	0	0	6(9)	1(1)	11.1	7(9)	3(3)	33.3
*Klebsiella*	*pneumoniae*	11(17)	1(1)	0	0	6(9)	1(1)	11.1	4(7)	0	0
	*oxytoca*	5(6)	2(2)	0	0	3(4)	1(1)	25	0	0	0
	Subtotal	16(23)	3(3)	0	0	9(13)	2(2)	15.4	4(7)	0	0
*Raoultella*	*planticola*	11(13)	7(9)	1(1)	11.1	2(2)	0	0	2(2)	0	0
	*ornithinolytica*	3(3)	1(1)	1(1)	100	2(2)	0	0	0	0	0
	Subtotal	14(16)	8(10)	2(2)	20.0	4(4)	0	0	2(2)	0	0
*Enterobacter*	*cloacae*	7(9)	2(2)	0	0	2(3)	1(1)	33.3	3(4)	2(3)	75
	*aerogenes*	2(2)	2(2)	0	0	0	0	0	0	0	0
	*asburiae*	2(2)	0	0	0	1(1)	1(1)	100	1(1)	1(1)	100
	*amnigenus*	1(1)	0	0	0	0	0	0	1(1)	1(1)	100
	Subtotal	12(14)	4(4)	0	0	3(4)	2(2)	50	5(6)	4(5)	83.3
*Pseudomonas*	*aeruginosa*	3(3)	0	0	0	3(3)	1(1)	33.3	0	0	0
	*fluorescens*	2(2)	0	0	0	2(2)	0	0	0	0	0
	*luteola*	1(1)	0	0	0	0	0	0	1(1)	0	0
	*putida*	1(1)	1(1)	0	0	0	0	0	0	0	0
	*stutzeri*	1(1)	1(1)	0	0	0	0	0	0	0	0
	Subtotal	8(8)	2(2)	0	0	5(5)	1(1)	20	1(1)	0	0
*Pantoea*	*Pantoea* spp.	6(7)	1(1)	1(1)	14.3	2(3)	1(1)	33.3	3(3)	3(3)	100
*Ewingella*	*americana*	3(4)	0	0	0	2(2)	1(1)	50	1(2)	0	0
*Acinetobacter*	*baumannii*	2(2)	0	0	0	0	0	0	2(2)	0	0
*Kluyvera*	*intermedia*	2(2)	0	0	0	2(2)	1(1)	0	0	0	0
Total		190(459)	79(224)	39(54)	24.1	60(120)	19(22)	18.3	51(115)	18(23)	20

Resistance breakpoints: *Aeromonas* and *Pseudomonas*, > 4μg/mL; others, > 2μg/mL.

Of the 206 isolates of *Aeromonas* spp., 117 *Aeromonas sobria (A. sobria)* isolates and 74 *Aeromonas hydrophila (A. hydrophila)* isolates were identified in 63 (20.3%) and 59 meat samples (19.0%), respectively (Table 1). In contrast, 100 *Yersinia enterocolitica (Y. enterocolitica)* isolates were identified in 57 meat samples (18.4%) and 39 *E. coli* isolates were identified in 27 meat samples (8.7%). In chicken meat samples, the isolation rates were as follows: *A. sobria,* 48.5%; *A. hydrophila,* 36.9%; *E. coli*, 18.4%; and *Aeromonas veronii (A. veronii)*, 11.6%. In pork samples, the isolation rates were as follows: *Y. enterocolitica*, 21.4%; *A. hydrophila*, 13.6%; and *A. sobria,* 9.7%. In beef samples, the isolation rate of *Y. enterocolitica* was 27.9% and the isolation rates of other bacteria were less than 10%.

### Colistin Resistance in the Isolated Bacteria

Of the 459 isolates, 99 (21.6%) isolates with MIC > 2 μg/mL and MIC > 4 μg/mL for *Enterobacteriaceae* and non- *Enterobacteriaceae* isolates respectively were resistant to CST (Table 1). These isolates included *Aeromonas* spp. (48/206, 23.3%), *Yersinia* spp. (5/112, 4.5%), *E. coli* (23/39, 59%), *Citrobacter* spp. (4/26, 15.4%), *Klebsiella* spp. (2/23, 8.7%), *Raoultella* spp. (2/16, 12.5%), *Enterobacter* spp. (7/14, 50%), *Pseudomonas* spp. (1/8, 12.5%), *Pantoea* spp. (5/7, 71.4%), *Ewingella* spp. (1/4, 25%), and *Kluyvera* spp. (1/2, 50%). CST-resistant bacteria were isolated from 39 of 103 chicken meat samples (37.9%), 19 of 103 pork samples (18.4%), and 18 of 104 beef samples (17.3%).

### Antimicrobial Resistance Profiles of the Isolated Bacteria

The antimicrobial resistance profiles of the dominant isolates are shown in Table 2. In 117 isolates of *A. sobria*, resistance to TET (29 isolates, 23.5%) was predominantly observed, followed by resistance to NAL (15 isolates, 12.8%), CHL (4 isolates, 3.4%), and CTX (2 isolates, 1.7%). In 74 isolates of *A. hydrophila*, resistance to CTX (1 isolate, 1.4%), TET (13 isolates, 17.6%), GEN (1 isolate, 1.4%), KAN (1 isolate, 1.4%), NAL (7 isolates, 9.5%), CHL (1 isolate, 1.4%), and SXT (1 isolate, 1.4%) was observed. In 100 isolates of *Y. enterocolitica*, resistance to TET was observed in 2 isolates (2%) and resistance to NAL and SXT was observed in 1 isolate (1%) each. In contrast, *E. coli* isolates from chicken meat samples showed resistance to AMP (17 isolates, 63.0%), CFZ (9 isolates, 33.3%), CTX (3 isolates, 11.1%), CAZ (2 isolates, 7.4%), TET (18 isolates, 66.7%), GEN (2 isolates, 7.4%), KAN (10 isolates, 37%), NAL (14 isolates, 51.8%), CIP (5 isolates, 18.5%), LVF (5 isolates, 18.5%), CHL (4 isolates, 14.8%), and SXT (7 isolates, 25.9%). In addition, *E. coli* isolates from pork samples were resistant to AMP (2 isolates, 40%), CFZ (2 isolates, 40%), CAZ (2 isolates, 20%), TET (2 isolates, 40%), NAL (1 isolate, 20%), CIP (1 isolate, 20%), LVF (1 isolate, 20%), and CHL (2 isolates, 40%), and *E. coli* isolates from beef samples were resistant to AMP (4 isolates, 57.1%), CFZ (2 isolates, 28.6%), TET (2 isolates, 28.6%), CHL (2 isolates, 28.6%), and SXT (2 isolates, 28.6%). All bacterial isolates were susceptible to MEM and AMK.

**Table 2.** Antimicrobial resistance profile of bacteria species to other tested antimicrobial agents

Bacteria	Meat type	Total isolate	Antimicrobial agents: Isolate No (% resistant isolates)													
			AMP	CFZ	CTX	CAZ	MEM	TET	GEN	KAN	AMK	NAL	CIP	LVF	CHL	SXT
***A. sobria***	C	101			2(2.0)	0	0	23(22.8)	0	0	0	14(13.7)	0	0	4(4.0)	0
	P	13			0	0	0	4(30.8)	0	0	0	1(7.7)	0	0	0	0
	B	3			0	0	0	2(66.7)	0	0	0	0	0	0	0	0
	Subtotal	117			2(1.7)	0	0	29(23.5)	0	0	0	15(12.8)	0	0	4(3.4)	0
***A. hydrophila***	C	44			0	0	0	9(20.5)	1(2.3)	1(2.3)	0	6	0	0	1(2.3)	1(2.3)
	P	18			1(5.6)	0	0	3(16.7)	0	0	0	0	0	0	0	0
	B	12			0	0	0	1(8.3)	0	0	0	1(8.3)	0	0	0	0
	Subtotal	74			1(1.4)	0	0	13(17.6)	1(1.4)	1(1.4)	0	7(9.5)	0	0	1(1.4)	1(1.4)
***Y. enterocolitica***	C	10			0	0	0	0	0	0	0	0	0	0	0	0
	P	34			0	0	0	2(5.9)	0	0	0	1(2.9)	0	0	0	1(2.9)
	B	56			0	0	0	0	0	0	0	0	0	0	0	0
	Subtotal	100			0	0	0	2(2)	0	0	0	1(1)	0	0	0	1(1)
*E. coli*	C	27	17(63.0)	9(33.3)	3(11.1)	2(7.4)	0	18(66.7)	2(7.4)	10 (37.0)	0	14(51.8)	5(18.5)	5(18.5)	4(14.8)	7(25.9)
	P	5	2(40)	2(40)	0	0	0	2(40)	0	0	0	1(20)	1(20)	1(20)	2(40)	0
	B	7	4(57.1)	2(28.6)	0	0	0	2(28.6)	0	0	0	0	0	0	2(28.6)	2(28.6)
	Subtotal	39	23(59.0)	13(33.3)	3(7.7)	3(7.7)	0	22(56.4)	2(5.1)	10 (25.6)	0	15(38.5)	6(15.4)	6(15.4)	8(20.5)	9(23.1)

Those printed in boldface are bacteria with intrinsic resistance to AMP and CFZ.

C, chicken; P, pork; and B, beef.

**Table 3.** Microbiological and resistance profiles of bacteria carrying the *mcr* genes

Location	Strain No.		Bacteria	Serotype	Multilocus sequence typing	Resistance profile	*mcr* gene	
							Subtype	Location
Kanto	CL-266	C	*E. coli*	O13/O135:H48	ST 10 (10-11-4-8-8-8-2)	CST	*mcr*-1.1	IncI2 Plasmid
	CL-276	C	*E. coli*	H34	ST2614 (31-276-83-140-1-187-2)	TET-CST-NAL-SXT	*mcr*-1.1	IncI2 Plasmid
	CL-304	C	*E. coli*	H31	ST101 (43-41-15-18-11-7-6)	AMP-CFZ-TET-CST- KAN-NAL-CHL-SXT	*mcr*-1.1	IncI2 Plasmid
Chubu	CL-21, CL-22	C	*E. coli*	H27	ST1112 (10-11-5-10-8-1-2)	TET-CST-KAN-NAL	*mcr*-1.12	IncI2 Plasmid
	CL-25, CL-26	C	*E. coli*	O91:H28	ST135 (13-39-50-13-16-37-25)	TET-CST	*mcr*-1.1	IncI2 Plasmid
	CL-480	C	*E. coli*	O81:H7	ST5826 (80-57-18-55-8-6)	AMP-TET-CST-NAL-SXT	*mcr*-1.1	IncI2 Plasmid
Kansai	CL-230, CL-231	C	*E. coli*	H52	novel (6-5-188-8-24-8-6)	TET-CST	*mcr*-1.1	IncI2 Plasmid
	CL-235	P	*A. sobria*			TET	*mcr*-3.25	Chromosome
	CL-184	C	*E. coli*	O91:H28	ST1196 (6-6-33-26-11-8-2)	AMP-CST-NAL-CPFX-LVFX	*mcr*-1.1	IncI2 Plasmid
Kyusyu	CL-859	C	*E. coli*	H5	ST206(6-7-5-1-818-2)	AMP-CST	*mcr*-1.1	IncI2 Plasmid
	CL-931, CL-933	C	*E. coli*	H5	ST206(6-7-5-1-818-2)	AMP-CST	*mcr*-1.1	IncI2 Plasmid
	CL-1133	C	*A. sobria*				*mcr*-3.25	Chromosome

Underlined strains were subjected to NGS analysis.

### Characteristics of *mcr*-harboring Isolates

Of the 365 isolates with CST MICs of ≥1 μg/mL, 16 harbored the *mcr* gene. In chicken meat samples, one *A. sobria* isolate from 1 sample (1.0%) and 14 *E. coli* isolates from 10 samples (9.7%) were positive for the *mcr*-3 and *mcr*-1 genes, respectively. In pork samples, one *A. sobria* isolate was positive for the *mcr*-3 gene. The CST MIC of two *A. sobria* isolates carrying *mcr*-3 was 1 μg/mL, indicating that the isolates were susceptible to CST.

Of the 16 isolates carrying the *mcr* gene, 12 were selected for next-generation sequencing (NGS) because only one isolate was selected when two were isolated from a sample. The serotypes, multilocus sequence typing results, bacterial subtypes, and the locations of the *mcr* gene and other resistance genes are shown in Table 3. NGS analysis revealed that plasmidic *mcr*-1.1 and *mcr*-1.12 were found in nine and one *E. coli* isolates, respectively, from chicken meat samples and chromosomal *mcr*-3.25 in two *A. sobria* isolates from pork and chicken meat samples. In addition, several acquired resistance genes to β-lactams (*bla*_TEM-1B_), tetracyclines (*tet*(A) and *tet*(B)), aminoglycosides (*aad*A5, *aph*(3”)-Ib, *aph*(3’)-Ia, and *aph*(6)-Id), phenicols (*cat*A1), quinolone (*qnr*S13), sulfonamides (*sul*1 and *sul*2), trimethoprim (*dfr*A1 and *dfr*A17), and macrolides (*mdf*(A)) were found in *mcr*-harboring *E. coli*. In addition, β-lactam resistance genes (*amp*S and *bla*_FOX-4_) and the TET resistance gene, *tet* (E), was found in one *A. sobria* isolate from pork samples.

**Table 3.** (Continued)

Strain No.	Resistance genes against							
	Aminoglycoside	β-lactam	Phenicol	Trimethoprim	Macrolide	Quinolone	Sulfonamide	Tetracycline
CL-266					*mdf*(A)			
CL-276	*aad*A5, *aph*(3”)-Ib, *aph*(6)-Id			*dfr*A17	*mdf*(A)	gyrA(S83L)	*sul*2	*tet*(B)
CL-304	*aph*(3”)-Ib, *aph*(3’)-Ia, *aph*(6)-Id	*bla*_TEM-1B_	*cat*A1	*dfr*A1	*mdf*(A)	gyrA(S83L)	*sul*1, *sul*2	*tet*(B)
CL-21, CL-22	*aph*(3”)-Ib, *aph*(3’)-Ia, *aph*(6)-Id				*mdf*(A)	gyrA(S83L)		*tet*(B)
CL-25, CL-26	*aph*(3”)-Ib, *aph*(6)-Id				*mdf*(A)			*tet*(A)
CL-480		*bla*_TEM-1B_		*dfr*A1	*mdf*(A)	gyrA(S83L)	*sul*1	*tet*(A)
CL-230, CL-231					*mdf*(A)	*qnr*S13	*sul*2	*tet*(A)
CL-235		*amp*S, *bla*_FOX-4_						*tet*(E)
CL-184		*bla*_TEM-1B_			*mdf*(A)	gyrA(S83L, D87N)		*tet*(A)
CL-859		*bla*_TEM-1_			*mdf*(A)			
CL-931, CL-933		*bla*_TEM-1_			*mdf*(A)			
CL-1133		*amp*S, *bla*_FOX-7_						

## 4. Discussion

The present study showed that domestic retail meats were contaminated with CST-resistant bacteria with or without *mcr* genes. The *mcr*-1 gene was detected in *E. coli* isolated from chicken meat samples (10/103, 9.7%) but not in *E. coli* isolated from pork or beef samples. In addition, chromosomal *mcr*-3 was found in *A. sobria* isolated from chicken meat and pork samples. In a previous study, an *E. coli* strain carrying the *mcr*-1 gene was found in domestic retail chicken meat (8/154, 5.2%) and imported retail pork (1/55, 1.8%) samples in Japan^24^^)^. The use of CST as a feed additive in food-producing animals, including poultry, has been prohibited since July 2018 in Japan; however, the therapeutic use of CST is approved in cattle and pigs. In the current study, the presence of the *mcr*-1 gene was observed in CST-resistant *E. coli* only from chicken meat samples. In Japan, the *mcr*-1 gene was detected in *E. coli* from healthy cattle (5/3,134, 0.2%), pigs (20/2,052, 1.0%), and broiler chickens (14/2,017, 0.7%) in 2000–2014^20^^)^. Despite the low prevalence of the *mcr*-1 gene, the frequent contamination of poultry carcasses with intestinal contents during the slaughter process has been reported^24^^)^. A study conducted in Bangladesh found high prevalence (25%) of the *mcr*-1 gene in *E. coli* isolated from broiler chickens, and this finding was associated with high CST usage for treatment and prophylaxis purposes^32^^)^. In addition, a survey in the Netherlands detected the *mcr*-1 gene (8%) in chicken^15^^)^. In the current study, more than 60% of the *mcr*-1 gene-containing bacteria isolated from chicken meat samples exhibited multidrug resistance. The contamination of meat with multidrug-resistant bacteria that can be transmitted to humans through meat consumption is a potential public health risk.

Furthermore, *A. sobria, A. hydrophila*, and *A. caviae* are some of the most important pathogens that cause gastroenteritis and wound infections in humans. In Japan, foodborne disease outbreaks caused by *A. hydrophila* or *A. sobria* are regulated by the Food Sanitation Act (Act No. 233 of 1947). CST resistance was observed in 48 *Aeromonas* spp. isolated from 32 chicken meat, 9 pork, and 7 beef samples, but these isolates did not carry the *mcr* genes tested. The *mcr*-3 gene was detected in two *A. sobria* isolates, from chicken and pork samples, with a CST MIC of 1 μg/mL. The *mcr*-3 gene was first reported in *E. coli* isolated from pigs^7^^)^ and has been reported to be responsible for CST resistance in *Aeromonas* spp. isolated from meat and environmental water samples^18^^,^^33^^)^. In Japan, current reports on the detection of the *mcr* gene from livestock and meat are limited to *E. coli* and *Salmonella*^20^^,^^21^^,^^22^^,^^23^^,^^24^^,^^34^^)^. However, because the *mcr* gene has been found in other gram-negative bacteria, further investigations on the prevalence of *mcr* genes in different bacteria are necessary.

Bacterial contamination of retail meats is an important issue that depends on various factors, including the differences in intestinal flora of animals and treatment processes in slaughterhouses^35^^)^. *Aeromonas* spp. and *E. coli* were frequently encountered in the present study, which is in accordance with food poisoning cases reported in Japan that were associated with *Aeromonas* spp. and *E. coli* isolated from chickens^36^^)^. Among the 14 CST-resistant *E. coli* isolates harboring *mcr*-1, some exhibited multidrug resistance, which can be attributed to the acquisition of resistance genes (Table 3). The CST-resistant *E. coli* isolates with the *mcr*-1 genes were susceptible to 3rd generation cephalosporins (CTX and CAZ) and carbapenem (MEM), suggesting that these antimicrobial agents may be an appropriate treatment for infections caused by these CST-resistant *E. coli* strains. However, there is a potential risk of horizontal transfer of the *mcr*-1 plasmid to other relevant *E. coli* possibly carrying extended spectrum beta-lactamase and carbapenemase resistance genes in humans. Hence, contamination of meat with such multidrug-resistant bacteria possessing *mcr* genes highlights the risk of resistance gene transmission to humans through meat consumption. Colonization of the human intestinal tract with multidrug-resistant bacteria can result in infections with limited treatment options.

The other gram-negative bacteria belonging to the *Enterobacteriaceae* family isolated in this study are part of the normal flora of the intestinal tract or environment of animals and have been associated with opportunistic infections in humans^37^^)^. In addition, *Y. enterocolitica* is a causative agent of food poisoning, is widely distributed in nature, and inhabits livestock, wild animals, water, and soil^38^^)^. Moreover, *Y. enterocolitica* serogroups O3 and O9 are pathogenic to humans^39^^)^, although the serogroups of the isolates were not investigated in this study. In this study, using CST-DHL media, we isolated CST-resistant bacteria among retail meat. However, we were not able to determine the mechanism underlying CST resistance in bacteria lacking the *mcr* gene. Therefore, further research is needed to elucidate the mechanism behind CST resistance in *mcr*-negative bacteria.

In conclusion, we detected CST-resistant bacteria in retail meats and *mcr*-1 and *mcr*-3 genes in *E. coli* and *A. sobria*, respectively. The findings of this study highlight the necessity of surveillance of CST resistance and resistance genes in bacteria that contaminate retail meats.
